# Visualizing the Global Landscape of Post-transplant Acute Lung Rejection

**DOI:** 10.7759/cureus.92662

**Published:** 2025-09-18

**Authors:** Niki R Patel, Meghana Konda, Latha Ganti

**Affiliations:** 1 Biology, Canterbury Collegiate Preparatory High School, Fort Myers, USA; 2 Public Health, Brown University, Providence, USA; 3 Medical Science, The Warren Alpert Medical School of Brown University, Providence, USA

**Keywords:** acute transplant rejection, bibliometric analysis, bibliometric mapping, bibliometric visualization, post-lung transplant complications

## Abstract

After lung transplantation, prior antibodies can rapidly attack the donor lung, causing damage and increasing acute rejection risk, specifically known as antibody-mediated rejection. While antibody matching has improved, numerous challenges remain in detecting and treating rejection. The lung’s exposure to its surroundings further activates immune responses. Persistent rejection and aggressive treatments can reduce survival rates. This review is a bibliometric analysis encompassing research on lung transplants and rejection published between 2016 and 2025. Insights were obtained by extracting elements from citation analysis from the Web of Science database and outlining emerging patterns and trends using VOSviewer (version 1.6.15; Leiden University, Leiden, The Netherlands). The analysis encompasses countries, institutions, publication years, and keywords that comprise the literature on acute rejection following lung transplantation.

## Introduction and background

Lung transplantation remains the definitive treatment for patients with end-stage pulmonary disease. In 2023, there were 3,049 adult lung transplants in the United States alone. However, acute rejection continues to pose a significant threat to graft survival and patient outcomes. Transplants have the ability to increase a patient's quality of life [1]. Two forms of acute rejection can occur after a lung transplant. Acute cellular rejection (ACR) is an immune response mediated by T cells that directly attack the donor organ. ACR involves the direct recognition of foreign proteins by cytotoxic T cells, which then trigger inflammation and cell death. The other form is antibody-mediated rejection (AMR), which involves antibodies attacking the organ's cells and blood vessels. AMR is characterized by donor-specific antibodies targeting donor-specific molecules on the graft [2]. Within minutes to hours of transplantation, pre-formed antibodies in the recipient may target donor human leukocyte antigens, resulting in endothelial injury and initiating a cascade of immune-mediated damage to the allograft [3]. While the implementation of improved screening and donor-recipient matching techniques has reduced the overall probability of rejection, management of acute rejection episodes remains challenging.

Antibody testing and matching have made rejection much less common. During the transplant procedure, exposure facilitates microbial invasion and macrophage activation, priming the innate immune response [4]. This increased immunologic reactivity contributes to the enhanced frequency and severity of acute rejection in lung recipients compared to other solid organ transplants [5]. The rate at which this ﬁeld progresses depends on the ability of researchers to overcome numerous challenges, including the number of donors, specification of candidates, and graft dysfunction [6].

If a lung transplant patient continues to experience rejection episodes, the function of their lung may decline rapidly. Additionally, due to the adverse eﬀects of the medications used for treatment, long-term survival rates of patients may decline [7].

Recently, scientists have discovered that the human body’s antibodies can attack the new lung, often leading to lung failure, which is known as AMR [8]. The consensus diagnostic criteria were created in order to ensure that precise standards are used for patients diagnosed with AMR. Despite the development of a consensus definition and diagnostic criteria for AMR, significant divergences remain in understanding its pathogenesis, subclinical forms, and optimal treatment strategies [9]. The acute rejection of a lung is crucial to discuss in order to portray research trends and assess research in relation to treatment strategies.

Although some progress has been made in the characterization and management of acute rejection, the current testing remains limited in its ability to provide robust, evidence-based intervention strategies. Emerging techniques and targeted immunomodulatory therapies oﬀer promising avenues for future investigation.

Since it has been established that disparities in treatment options for acute rejection after a lung transplant are eminent, this bibliometric analysis aims to identify the trends in current literature to depict the imperative need for greater research on rejection after a lung transplant.

## Review

Data source and search strategy

This study used data from the Web of Science (WOS) Core Collection, a comprehensive database containing high-impact scientific publications dating from the early 1900s to the present. The WOS Core Collection is widely regarded as a reliable source for bibliometric analysis, due to its extensive coverage of peer-reviewed literature and robust citation indexing [10]. The search focused on literature related to lung transplantation and rejection, covering the publication period from 2016 to 2025. The search strategy was restricted to the “topic” field in WOS, which includes article titles, abstracts, and keywords. The specific search phrase used was “lung transplant and rejection,” without the application of any filters for punctuation, capitalization, article type, or language, ensuring that all relevant documents were included regardless of format or linguistic origin. The exact search string was: ((TS=("lung transplant") OR TI=("lung transplant")) AND (TS=("acute rejection") OR TI=("acute rejection"))) AND PY=(2016-2025). Inclusion criteria were any article type within these search parameters. In order to obtain the most comprehensive visualization of the literature, no exclusion criteria were applied. All documents from this search were included in the analysis. Since only a single database (WOS) was used, there were no duplicates. Search results were exported in batches of up to 500 records as text-delimited ﬁles, including the “full record” for each article. 

Data analysis

This study used a bibliometric analysis method to quantitatively assess the body of literature related to lung transplant rejection and associated therapeutic strategies. Bibliometric analysis oﬀers a systematic approach to evaluating research output, citation patterns, and thematic evolution within a given scientific ﬁeld [11]. Delimited text ﬁles extracted from the WOS Core Collection were imported into VOSviewer (version 1.6.15; Leiden University, Leiden, The Netherlands) for processing and visualization. Key bibliographic data, including author names, article abstracts, keywords, journal sources, and citation counts, were gathered from each record to map trends and research productivity from 2016 to 2025. The computer mapping software, VOSviewer, was then employed to identify the most prominent contributors in the ﬁeld, including frequently cited authors, high-impact journals, and most commonly used keywords.

Minimum occurrences were set at 5 to optimize the number of nodes visualized, without sacrificing too many infrequently occurring ones. Co-authorship was adjusted to 25. Given the niche topic of this review, keywords were limited to 100 to make the clusters easier to visualize. Full counting was used for keyword co-occurrence to identify all topics covered. Fractional counting was used for institutions and countries to obtain a balanced view of contributions.

These data points were used to generate bibliometric maps that illustrate bibliographic coupling, highlighting how authors, journals, and publications are interlinked through shared citations [12]. The visual maps produced by VOSviewer display the frequency and strength of connections among terms such as authors, countries, journals, and keywords. These relationships are represented by linking lines, where the length and thickness of each line denote the intensity of co-occurrence or citation linkage [13].

Results

**Figure 1:**
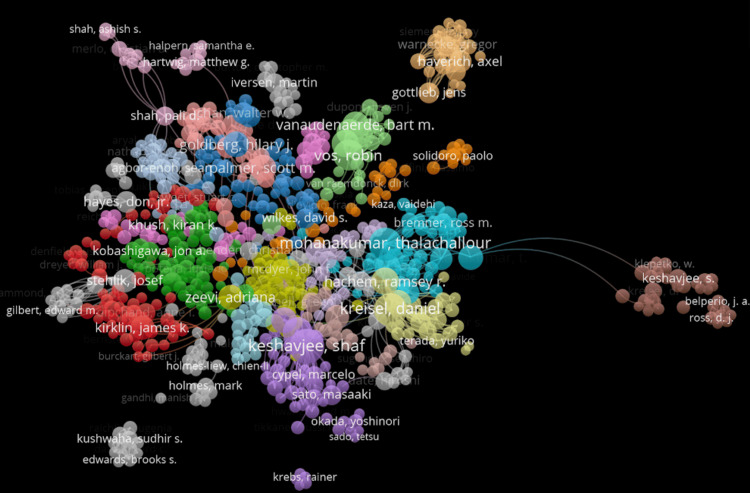
Distribution and frequency of authors based on their collaborative relationships regarding acute rejection following lung transplant

Figure 1 displays the distribution and frequency of authors based on their collaborative relationships regarding acute rejection succeeding lung transplant.

It demonstrates a clustering of authors based on co-authorship in the domain of lung rejection research, The color-coded nodes suggest strong collaboration. Notably, authors such as Keshavjee, Shaf, and Thalachallour appear as central nodes, emphasizing their significance. Other dense clusters include those of Stehlik and Kreisel. The separation of some clusters such as those of Keshavjee and Kirk suggests emerging research. Overall, this visual reflects a group of highly connected authors.

**Figure 2:**
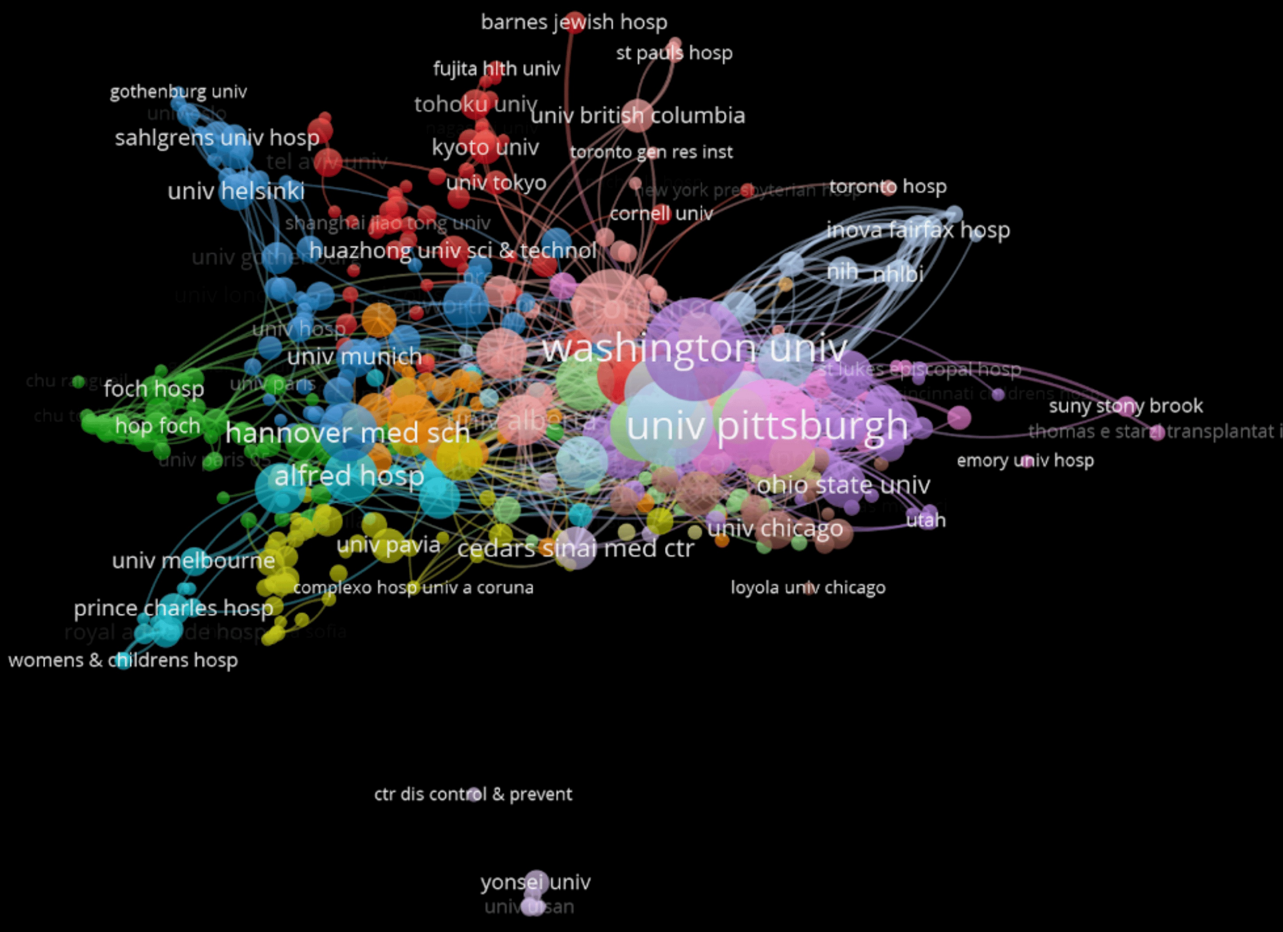
Distribution and frequency of published papers of varying organizations regarding lung transplant and rejection

Figure 2 depicts clusters of organizations involved in lung rejection research, with collaboration patterns shown by connections. The University of Pittsburgh and Washington University are the most central and densely packed, suggesting a strong influence in the field. Yonsei University appears to be isolated, possibly due to geographical distance or limited international partnerships. While global research exists, partnership is largely concentrated within the United States. 

**Figure 3:**
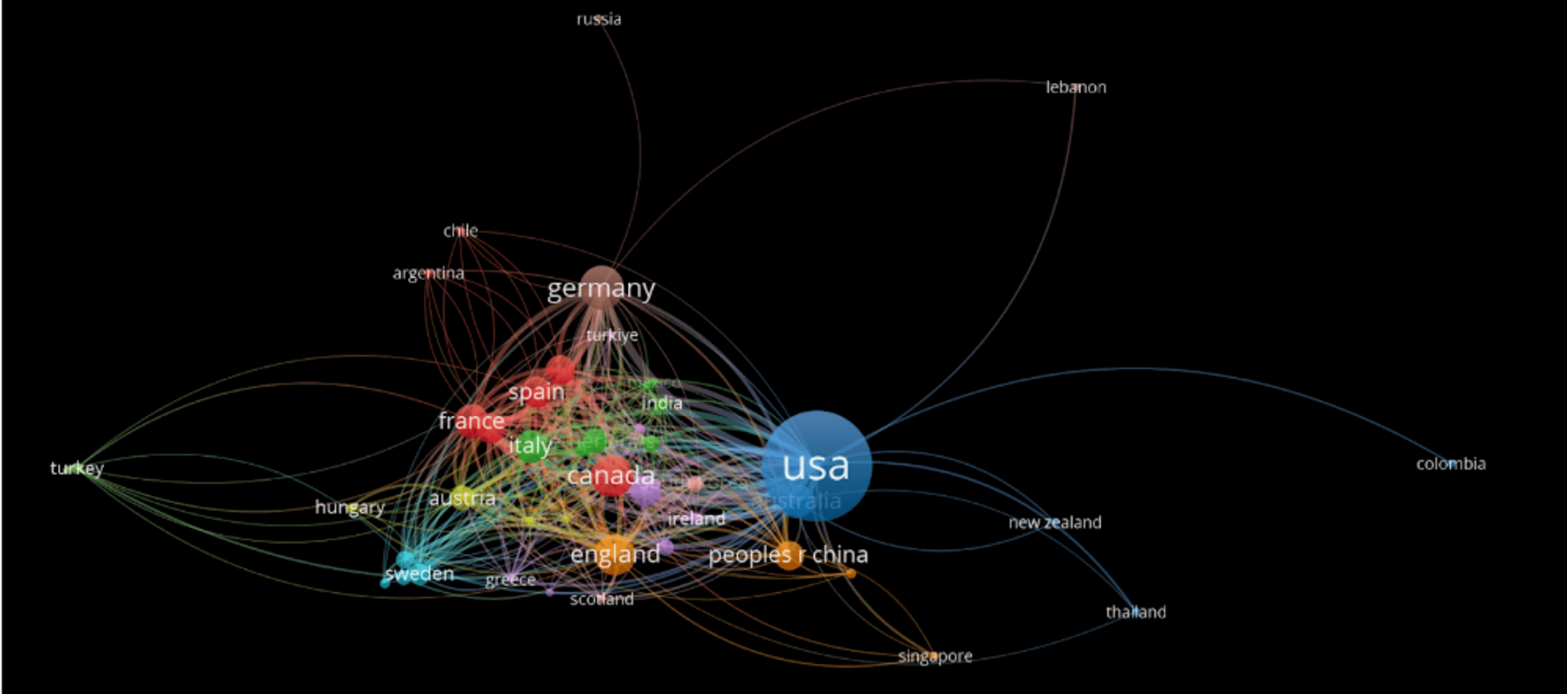
Distribution and frequency of published papers in different countries regarding lung transplant and rejection

Figure 3 showcases the global distribution of lung transplant and rejection research. The US leads, with visible links to countries such as Germany, Canada, England, Spain, France, and Italy. In contrast, other nations such as Singapore, Russia, New Zealand, Thailand, Colombia, and Lebanon appear more isolated, with limited ties, indicating research and publication efforts that are less integrated into their regions.

**Figure 4:**
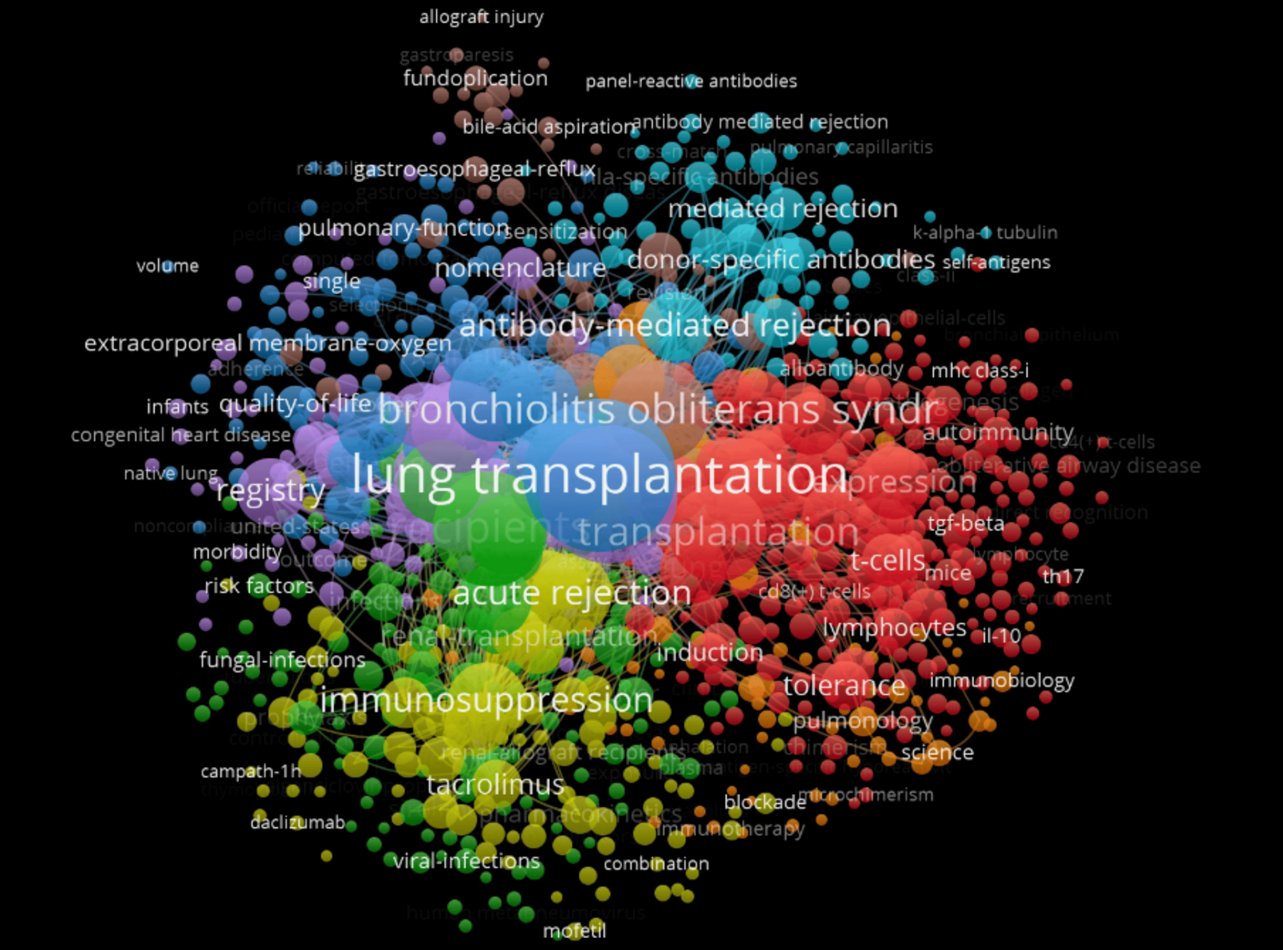
Most common keywords used in relevant articles published between 2016 and 2025

It appears that the most frequently used keywords in lung transplants and rejection articles from 2016 to 2025 include lung transplantation, AMR, bronchiolitis obliterans syndrome (BOS), acute rejection, nomenclature, and immunosuppression, indicating major research focuses. Conversely, less frequent terms such as volume, k-alpha tubulin, mofetil, combination, morbidity, risk factors, extracorporeal membrane-oxygenation, and autoimmunity stipulate more niche or emerging topics within the field (Figure 4).

**Figure 5:**
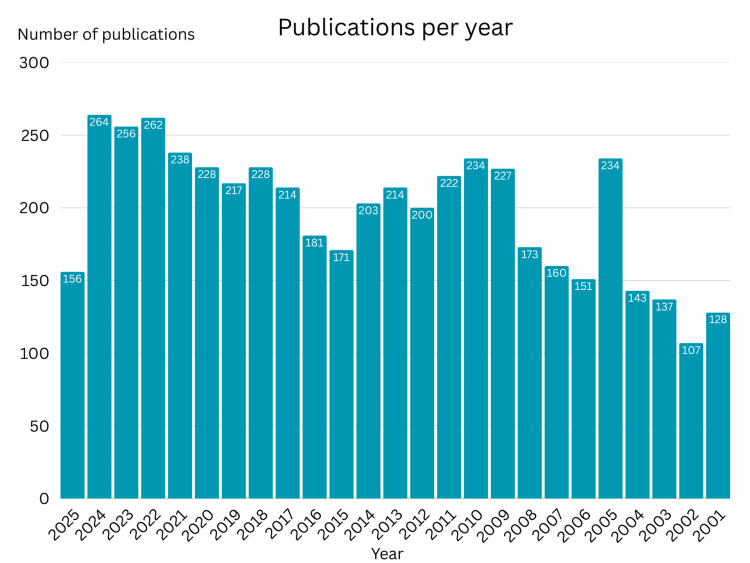
Chronological distribution of the publication years of the number of published papers regarding lung transplants and rejection in the past 25 years

The data reveal a rise in publications from 2001 to 2025, with peaks in growth around 2005 and continuing through the 2010s. There are also peaks in 2021-2023, with over 250 publications each year, suggesting increased research, possibly due to increased awareness from COVID-19 and an interest in respiratory health. The drop in 2025 is due to incomplete data for the current year rather than an actual decline in research. The visual begins with 128 papers in 2001 and ends with 112 in the year 2025 (Figure 5).

**Figure 6:**
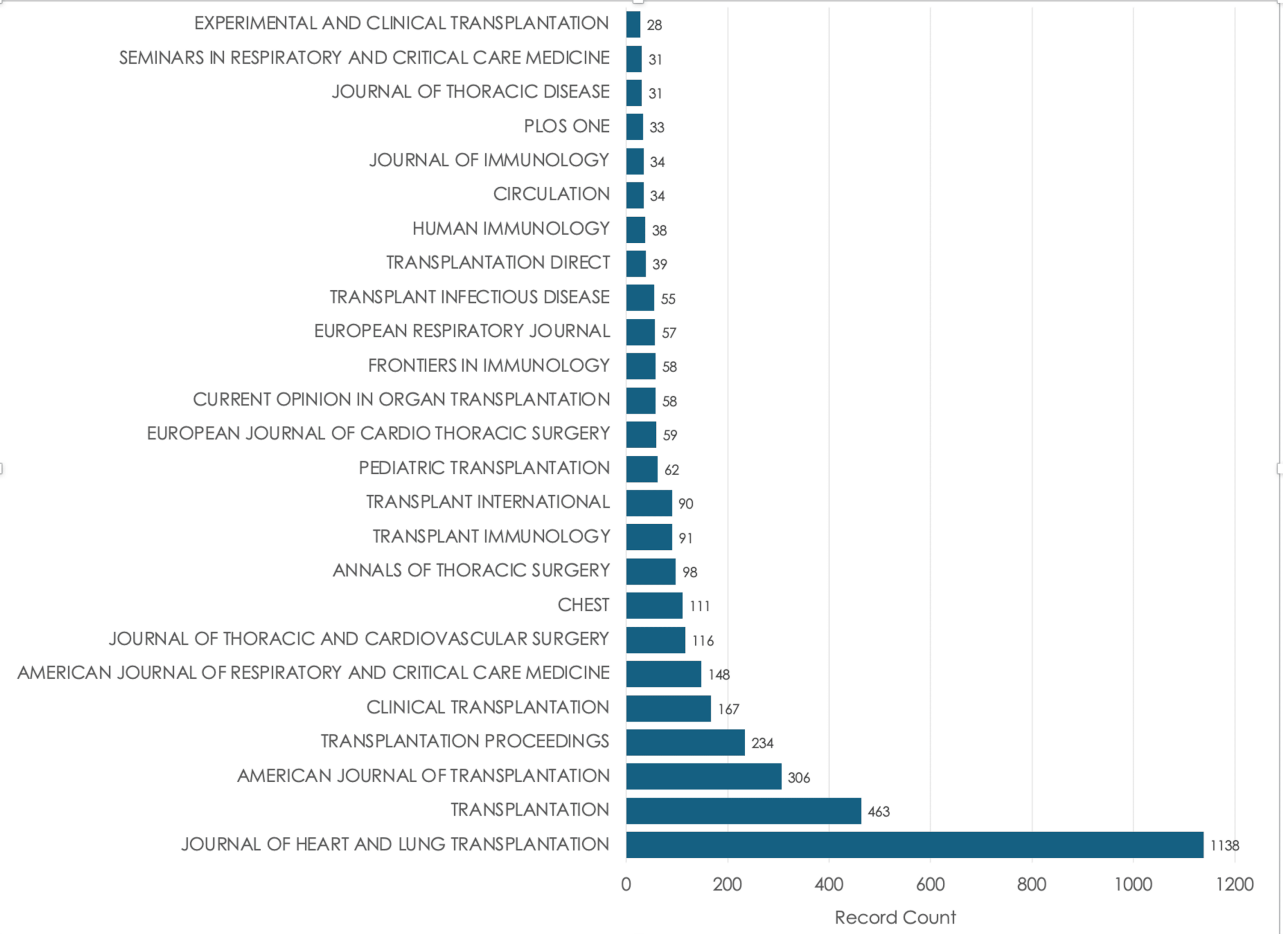
Distribution of published articles by journal

The Journal of Heart and Lung Transplantation leads with 1,133 publications, while Experimental and Clinical Transplantation shows the fewest at 26. A total of 3,557 out of 6,012 articles are represented in this figure. Transplantation consists of 462 publications, and the American Journal of Transplantation has 303. On the contrary, journals such as the Journal of Thoracic Disease and Seminars in Respiratory and Critical Care Medicine each have around 30 published articles, showing lower but still contributions that are accounted for (Figure 6).

Discussion

Acute rejection following lung transplantation remains a critical area of study, particularly as significant challenges persist in its treatment. This bibliometric analysis offers insight into the global research field on lung transplantation and acute AMR from 2016 to 2025. By evaluating collaborative networks, publication trends, and keywords, this study highlights the current focus areas, patterns, and notable variants in the literature. Special emphasis is placed on AMR, a particularly severe and complex form of rejection, to better understand its growing research profile.

The author collaboration network displayed in Figure 1 reveals clusters of researchers with varying degrees of connectivity. Larger, more interconnected clusters suggest broad collaboration and shared focuses, while smaller or isolated clusters such as the one surrounding Kirk may represent more specialized or less integrated research efforts. These isolated nodes, while less connected, may still offer valuable and novel insights, indicating opportunities for expanded interdisciplinary collaboration.

The data depicted in Figures 2, 3 reveal that the United States appears to be the most densely packed with the most connections, indicating that the University of Pittsburgh and Washington University have contributed a great deal of advancements to the field through their research. This is coherent due to their funding and the amount of resources the US receives compared to other countries. In contrast, countries such as Singapore, Thailand, and Colombia are represented by limited and loosely connected outputs, reflecting disparities in global research participation. Encouraging cross-national collaboration, particularly with underrepresented regions, may help bridge this gap and drive innovation in AMR diagnostics and treatment [14].

The data shown in Figure 4 depicts commonly used keywords, while those with lower frequencies suggest underexplored areas of research that may offer valuable information for overcoming current limitations in the field. The keywords include BOS and immunosuppression. In fact, BOS is one of the main reasons for fatality after a transplant, where there is irreversible airway obstruction [15]. Despite rising input that immune responses to lungs contrast with other organs, immunosuppression in lung transplants stems from treatments exhibited by recipients of other grafts [16].

The statistics shown in Figure 5 show a spike in the years 2021-2023, likely due to a newfound interest in respiratory disease due to COVID-19. The decline in 2025 is due to the unavailability of data. Figure 6 displays the central journals that publish articles on topics related to lung transplants and rejection. Identifying these journals helps find where innovative findings are being published, which can guide researchers toward trusted sources and steer them away from false information.

One limitation of this analysis is that the graphs may not fully express the accuracy of the connections. Additionally, terms with multiple meanings might be grouped together, which could lead to deceptive associations. Further, the data is only as reliable as what has been recorded in the database, and other records may not appear [17]. Isolated researchers could be providing valuable insight that is not being reflected due to their lack of connections. This also raises the concern that lesser-known publishers may face challenges in gaining influence, no matter the significance of their research. The fact that only a few journals have published hundreds or thousands of articles shows that the field is highly centralized, and there are lesser-known journals that should be looked into.

Acute AMR remains one of the most serious issues to lung transplant survival, and this bibliometric analysis reflects the urgency of its confrontation [18]. From 2016 to 2025, research in this area has grown exponentially, with most publications about immune-related complications. AMR’s hidden symptoms require enhanced diagnostic approaches [19]. The peaks in publication during 2021 to 2023 reveal a broader interest in respiratory health after COVID-19. This analysis not only tracks the current state of lung transplant research but also emphasizes gaps in the global communication surrounding acute AMR.

## Conclusions

This bibliometric analysis reveals that the most frequent keywords were lung transplantation, AMR, bronchiolitis obliterans, and immunosuppression. The most prominent authors in this research domain include Drs. Keshavjee, Shaf, and Thallachour. The United States dominates in the number of publications, with the two most productive institutions in this space being the University of Pittsburgh and Washington University. 

This study examines how acute lung transplant rejection has been researched over time. It displays who has been researching, the gaps in knowledge, and the connectivity between authors and institutions. While progress has been made, there is still a protracted path for figuring out superior treatments and comprehending the hidden forms of acute AMR.
